# Covalent Organic Frameworks as Nanocarriers for Improved Delivery of Chemotherapeutic Agents

**DOI:** 10.3390/ma15207215

**Published:** 2022-10-16

**Authors:** Weiming Liu, Xinyu Ma, Shuayb Mohamed Kheyr, Anjie Dong, Jianhua Zhang

**Affiliations:** 1 Key Laboratory of Systems Bioengineering of the Ministry of Education, Department of Polymer Science and Engineering, School of Chemical Engineering and Technology, Tianjin University, Tianjin 300350, China; 2 Tianjin Key Laboratory of Membrane Science and Desalination Technology, Tianjin University, Tianjin 300350, China

**Keywords:** covalent organic frameworks, nanocarriers, drug delivery, nanomedicine

## Abstract

Cancer has become one of the main causes of death worldwide. Chemotherapy as one of the main therapy modalities is very unsatisfactory. The various nanocarriers have brought new opportunities for effective tumor treatment. However, most of the current nanocarriers still suffer from low efficiency and confront significant challenges in overcoming multiple biological barriers. Compared with conventional nanocarriers, covalent organic frameworks (COFs) with unique and attractive features exhibited great potential to serve as a promising platform for anticancer drug delivery. In this review, we first summarize the strategies and challenges of nanocarriers for cancer chemotherapy and then highlight the recent advances in COF-based nanocarriers for improved delivery of chemotherapeutic agents. Finally, the challenges remaining for COF-based nanocarriers for clinical applications are outlined.

## 1. Introduction

Cancer is one of the leading causes of human death worldwide. In 2020, there were about 19 million new cancer cases and around 10 million deaths all over the world. Surgery was the mainstay of cancer treatment modality, especially for the early stage. For treating the tumors in the mid and late stages, several therapeutic strategies, including chemotherapy, radiation therapy, phototherapy, and sonodynamic therapy, were used [1]. The improved treatment accelerated progress against various cancers, leading to a distinct drop in overall cancer mortality. The death rate of cancer has decreased incessantly and achieved a total decline of about 30% in the last several decades [2]. However, these treatment strategies still face a large number of challenges in order to enhance the efficiency of treatment and ultimately cure the cancers.

**Figure 1:**
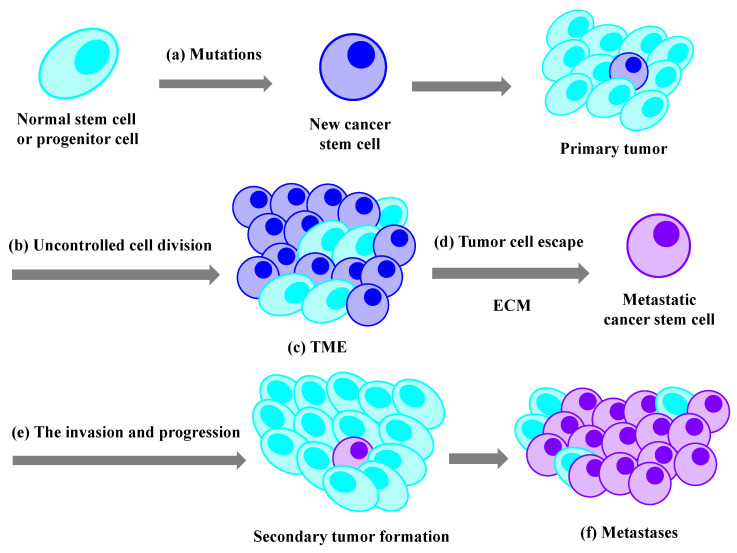
Main steps in the progress of cancer. (**a**) Tumorigenesis; (**b**) uncontrolled cell growth and division; (**c**) TME; (**d**) extravasation from the original tissues; (**e**) the invasion and progression; (**f**) the creation of cancer cell niches (metastasis).

Among the various therapeutic methods for tumors, chemotherapy based on using chemotherapeutic agents to kill tumor cells has become one of the most widely applied treatments all over the world. As a drug treatment, the successful delivery and utilization of chemotherapeutic drugs plays a key role in strangling the fast-growing malignant tumor cells. Many different chemotherapeutic drugs are available, but most of them still suffer from some key issues. For example, their poor water solubility causes the main barrier to their clinical use, especially for intravenous injection, and the fast blood clearance leads to insufficient bioavailability. In addition, the fast metabolism in vivo and the undesirable adsorption of anticancer drugs often result in serious side effects and systemic toxic effects [3,4]. Moreover, due to the non-specific distribution of anticancer drugs in the whole body, most chemotherapeutic drugs after administration distribute throughout the entire body and thus cause serious systemic side effects [5]. In addition, it is well known that cancer is a disease in which some tumor cells in the body grow uncontrollably and then spread into healthy parts of the body or invade nearby tissues. The process of normal cells transforming into tumor cells is typically a multi-stage process. The tumorigenesis process is shown in Figure 1 [6]. The tumor tissues are not only a cluster of tumor cells, but they are complex and heterogeneous environments consisting of resident host cells, an extracellular matrix, and secreted factors, as well as neoplastic blood vessels. This kind of tumor microenvironment (TME) forms and promotes angiogenesis and metastasis. In addition, the blood vessels in TME are an aberrant and disorganized vasculature. This kind of unique TME also serves as an intractable biological obstacle to anticancer drug delivery. As a result, only a small fraction of the drugs can reach the tumor [7,8,9].

## 2. Nanoparticles for Chemotherapy

Nanoparticulate drug delivery systems have presented great benefits and been generally exploited in the fields of antitumor drug delivery. In the last several decades, thousands of different nanoparticles (NPs) have been developed and used as drug delivery platforms for cancer treatment [10,11,12,13]. The NPs mainly include organic NPs and inorganic NPs. The organic NPs can be classified into liposomes, nanoscale micelles from self-assembly of amphiphilic polymer chains, nanogels from crosslinked hydrophilic polymer chains, and NPs derived from dendrimers. The inorganic NPs mainly include silica NPs and graphene-based nanocarriers, as well as metal and metallic oxides NPs. Compared with conventional chemotherapy, various NP-based treatments can significantly enhance the efficiency of drug delivery and drug retention at targeted site, thus reducing side effects for patients [14,15,16]. According to the latest research reports, about 100 nanomedicines based on various nanocarriers have been approved for commercialization by the FDA. Moreover, over 150 nanotechnology-based drug products are presently under clinical trials [17,18]. Amongst the approved nanomedicines, about one-third of nanomedicines were used for cancer treatment. Some typical nanomedicine products that are available for clinical use in oncology are summarized in Table 1. The increasing number of cancer-related nanomedicines approved for commercialization demonstrates the enormous opportunities and urgent requirements of nanoparticulate drug delivery systems for better cancer treatments.

However, most of the current NPs are still far from acceptable at the clinical level. For example, the liposomes and polymer micelles with great promises for pharmaceutical applications are formed by the self-assembly process, which still suffers from poor storage stability, dilution-induced destabilization during blood circulation, undesirable drug leakage, and uncontrolled drug release at the targeted site. To balance the dilemma between the stability during blood circulation and the fast drug release within targeted tumor cells, the polymeric NPs or nanogels with crosslinked cores or shells have been demonstrated to be able to increase the stability and reduce the burst release during blood circulation. Nevertheless, the crosslinking would severely inhibit the release of drugs at the target sites [19]. The inorganic or metal NPs exhibited some desirable properties for drug loading and delivery, such as high drug loading efficiency, chemical/thermal stability, and scalable preparation as well as flexibility in functionalization. However, their applications are often hindered by their poor biodegradability and undesirable toxicity as well as a very high foreign body response or inflammatory response.

To achieve ideal delivery efficacy, the physicochemical properties and nanostructures of the nanocarriers, including shapes and sizes, elasticity and stiffness, porosity, and high surface-to-volume ratio, as well as surface charge, etc., should be fully taken into consideration toward investigating their effect and interaction with biological systems. From the injection site to the targeted tumor cells, the nanomedicines will confront the sequential drug delivery barriers, fast clearance of blood circulation, extravasation from the blood vessels at the tumor site, enhanced penetration into the deep part of tumors, effective internalization, and controlled drug release inside the tumor cells. Currently, no nanocarriers can simultaneously meet the contradictory requirements to overcome the sequential barriers, hindering their translation into clinical medicine. As a result, various strategies, mainly including surface hydrophilic modification, passive and active targeting modification, as well as stimulative responsibility, have been designed and endowed the functionalized NPs with advanced and intelligent functionalities over the last several decades [20].

## 3. Functionalization Strategies of Nanocarriers

**Figure 2:**
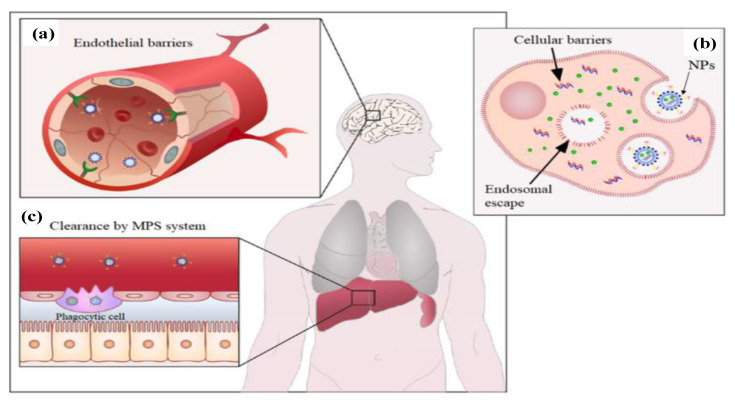
Main physiological barriers faced by nanoparticles (NPs). (**a**) Endothelial barriers, during the extravasation process into tumor tissues. (**b**) The endo-lysosomal compartment, during the absorption of NPs by target cells. (**c**) The clearance of NPs by phagocytic cells, especially the mononuclear phagocyte system (MPS) [21]. Reprinted with permission from ref. [21]. Copyright 2018 Springer Nature.

The applications of NPs for drug delivery have achieved great achievements and shown enormous advantages. However, the efficient delivery of chemotherapeutic drugs to target cells was still severely restricted by various anatomical and physiological barriers. As shown in Figure 2, the main obstacles mainly include the fast uptake and clearance by the mononuclear phagocytic system during blood circulation, their interaction with plasma proteins, the intrinsic characteristics of TME, efficient distribution into tumor tissues, tumor penetration, and cellular internalization, as well as intracellular drug release [21]. Consequently, the NPs were designed and functionalized in order to achieve active and specific targeting for promoting the preferential accumulation in tumor tissues, effective uptake by tumor cells, and release of the drug after entering cancer cells [22,23].

### 3.1. Passive Targeting Strategies

**Figure 3:**
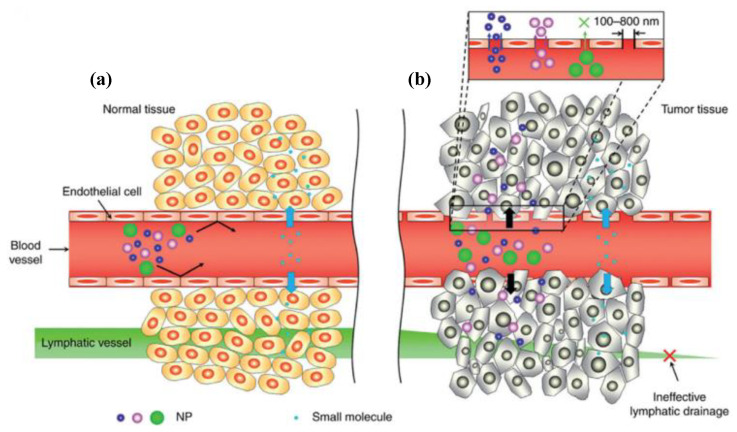
Physiopathological characteristics and passive accumulation of NPs and small molecules in (**a**) healthy tissues (≤8 nm) and (**b**) tumor tissues (40 nm to 1 μm), mainly due to the EPR effect [25]. Reprinted with permission from ref. [25]. Copyright 2017 Elsevier.

The unique TME and the pathophysiological characteristics of tumor tissues are one of the main barriers to drug delivery [7,9]. However, the physicochemical characteristics and unique nanostructures of NPs can be preferably accumulated at the tumor site by the enhanced permeability and retention (EPR) effect [24]. As shown in Figure 3, the preferential accumulation of NPs in tumors is because solid tumors usually possess several pathophysiological characteristics: the hyper vascularization and secretion of vascular permeability factors that allow NPs to translocate from the blood compartment to the perivascular TME, and thus cause enhanced permeation t [25]. Other pathophysiological characteristics of solid tumors are their abnormal form, characterized by fenestrations, the rough muscle layer, and the absence of effective intratumor lymphatic drainage. When the drug-loaded NPs are extravasated into the interstitial space of the tumor, the NPs’ diffusion back into the bloodstream is usually prevented, as their flow rate to the exterior bloodstream of the tumor is slowed down due to the dense collagen matrix. Thus, the drug-loaded NPs are retained in the interstitium for a longer time, leading to effective targeting of the tumor [26], wherein the drug is released through enzymatic degradation and internalized by the cells through passive diffusion [8]. A nanosystem size between 10 and 200 nm is suitable for an efficient and non-damaging treatment since NPs with sizes of over 200 nm can be easily trapped by the liver and spleen, which belong to the reticuloendothelial system (RES) and are often associated with the mononuclear phagocyte system (MPS). In the same way, NPs with a size below 8 nm are quickly cleared by renal filtration [27]. However, size is not the only factor that influences the EPR effect, but also the shape of the NPs plays an important role in the diffusion throughout the tumor tissue. For example, some nanorods have exhibited more rapid tumor penetration than nanospheres [28,29], thus enhancing tumor penetration and accumulation.

**Figure 4:**
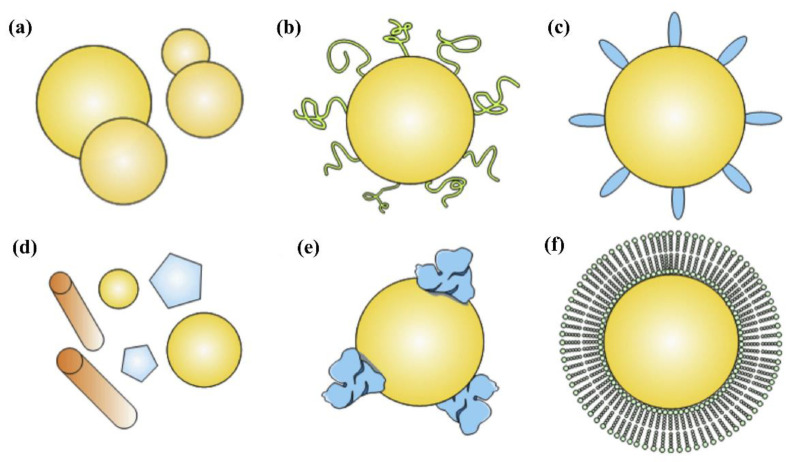
Strategies to prolong the half-life of PNs in the circulatory system by adjusting their physical characteristics, such as (**a**) size and (**b**) shape; or by (**c**–**f**) surface chemistry including (**c**) PEGylation and coating of PNs with (**d**) human serum albumin, and “self” markers such as (**e**) peptides or (**f**) cell-based membranes extracted from leukocytes and red blood cells [32]. Reprinted with permission from ref. [32]. Copyright 2017 Elsevier.

Tumor heterogeneity and intrinsic features of TME, such as hypoxic gradient, acidosis, high interstitial fluid pressure, and low microvascular pressure, can limit the delivery of nanomedical drugs to the tumor by passive targeting, thus affecting their efficacy [30]. In addition, the delivery efficiency and the resultant therapeutic efficacy are obviously determined by the blood circulation time of NPs. After entering the human body, NPs inevitably contact a huge variety of biomolecules, such as sugars, proteins, and lipids. The nano-bio interactions lead to the formation of the so-called “protein corona” on the outer surfaces of NPs [31]. As shown in Figure 4, the transport behavior of NPs in blood and the clearance by the reticuloendothelial system and/or mononuclear phagocytic system are dictated by the physicochemical properties of NPs, such as particle size, particle shape, and surface feature [32]. To achieve good dispersion in serum, NPs are mostly coated with biosafe surfactants, hydrophilic polymers, and biodegradable copolymers, especially polyethylene glycol (PEG). The surface conjugation of PEG can significantly suppress the interactions between NPs with blood components, such as plasma proteins and enzymes. As a result, the circulation time of NPs are prolonged significantly and thus improve the EPR effect and enhance therapeutic results [26]. Despite the great success of PEG-conjugated substances, an unexpected immunogenic response of PEG (accelerated blood clearance phenomenon) has been extensively observed during the repeated administration of PEGylated nanocarriers, leading to increased clearance. Recently, cell membrane biomimetic NPs were widely designed and some special functions were realized, such as the prolonged blood circulation time, immune escape, and specific recognition. Generally, the human serum albumin or red blood cell membrane, as well as the macrophage plasma membrane, were extracted from leukocytes, red blood cells, and macrophage plasma, which were used as “self-markers” to coat onto the outer surface of various NPs. The biomimetic NPs can inherit the unique biofunction of the cell membrane to prevent recognition and clearance [33,34].

### 3.2. Active Targeting Strategies

**Figure 5:**
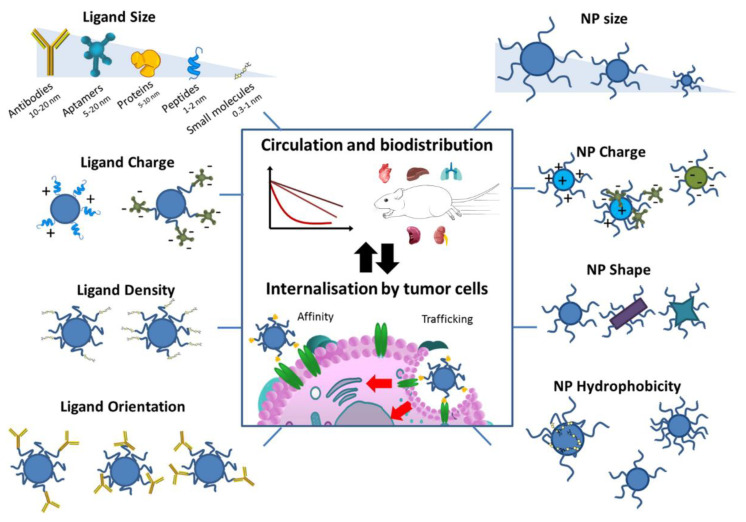
Physicochemical properties of ligands and NPs affecting blood circulation profiles, bio-distribution, and the internalization of ligand-functionalized nanocarriers [42]. Reprinted with permission from ref. [42]. Copyright 2014 Elsevier.

The past several decades have witnessed great progress in passive nanoparticle targeting strategies. As an important concept in nanomedicine, some passively targeted NPs, for example, long-circulating liposomes, have been approved by FDA for the treatment of solid tumors. However, the passive targeting strategies just via the EPR effect cannot ensure complete targeted delivery. Due to the complex biological barriers within the body, some studies have confirmed that most of the administered NPs were found to be captured and removed by mononuclear phagocyte systems, thus ending up in non-target sites. A recent analysis reported that only about 1% of the injected NPs dose reaches the tumor. Most of the administered NPs were found in healthy organs such as the liver and spleen. The amount of NPs accumulated within the targeted tumor is insufficient to induce an antitumor response [35]. In contrast to passive targeting strategies, active targeting NPs with specific surface ligands can recognize and bind to specific cell surface receptors on cancerous cells [36,37]. The active targeting ligands, including peptides, proteins, nucleic acids, aptamers, sugars, and small molecules such as folic acid and carbohydrates have been widely used to functionalize NPs [38]. The ligand-modified NPs can increase the specific interaction with targeted diseased cells [39]. As a result, this kind of strategy can improve the affinities of NPs to tumor cells, increase the retention of NPs at diseased sites, and promote the uptake of NPs by cancer cells, leading to a significant improvement in the delivery efficacy and an obvious decrease in the toxicity of therapeutic agents. For example, folate is a vitamin with a high affinity for the folic acid receptor, overexpressed in the TME and certain tumors from 100 to 300 times above endogenous levels, so several folate-modified strategies have been widely developed by the synthesis of folic acid–drug conjugates or the grafting of folic acid onto the outer surface of nanocarriers. In addition to the active targeting of receptor overexpressed in tumor cells or tumor tissues, the tumor vasculature is a viable drug target. The tumor vasculature is not simply blood vessels. Compared with normal vasculature, tumor vasculature generally has a disorganized structure, abnormal cell adhesions, and basement membrane structures, as well as an especially inefficient blood supply. Clearly, the tumor vasculature is significantly different from the normal vessels. Therefore, the active targeting of the tumor vasculature, generally on the basis of the positive and negative regulation of angiogenesis/lymphangiogenesis, has been widely used as a novel strategy to enhance the accumulation of drug-loaded NPs into tumors, especially in combination with antitumor therapy along with anti-angiogenic therapies [40]. In addition to the current extracellular targeting strategies as mentioned above, the intracellular targeting strategies, especially the nuclear targeting strategy, can also increase the therapeutic efficacy by minimizing off-target effects. This kind of targeting strategy is dependent on specific peptide signals, which can interact with organelle receptors such as the nucleus, mitochondrion, and peroxisome through passive diffusion and active transport, allowing for the controlled release of the payload in the appropriate subcellular location [41]. As shown in Figure 5, the properties of the ligands concerning size, density, orientation, charge, and affinity must be taken into account [42]. Since NPs’ avidity is directly related to ligand density, achieving high ligand surface density and low nanocarrier opsonization is the main goal not only to attain high targeting efficiency but also to increase cell binding, and ensure optimal internalization and improve drug accumulation in tumors when studied in vivo [43]. Furthermore, the phenomenon called the “binding site barrier” at the tumor periphery is crucial to create ligand–receptor interactions strong enough to reach the targeting sites but without hampering the penetration of functionalized nanocarriers into the interstitial space [40]. Hence, the binding of the appropriate ligands on the NPs surface is of essential importance for designing active target systems. Strategies for ligand attachment are based on either covalent bonding or physical adsorption using affinity complexes. In the former, the ligands, mainly small molecules, are either bound to the NPs before assembly (pre-formulation strategy), or they can also be bound by reacting with formulated NPs (post-formulation conjugation), involving all types of ligands, preferably those that are not stable in organic solvents, have a large size, or have physicochemical properties that are not compatible with copolymers. In addition, “click chemistry” could be a useful synthetic method for conjugation, which involves a one-step reaction of heteroatom bonds with or without catalysts.

Despite the growing potential of nanomedicine in the field of site-specific delivery based on various targeting strategies, brain-targeted drug delivery still presents a tremendous challenge to scientists, as the existence of the blood–brain barrier (BBB) can dramatically impede the systemic delivery of pharmaceuticals from the blood into the brain. Intensive research has been devoted to developing promising and attractive strategies to enable nanomedicines or therapeutics effectively circumvent BBB. Active targeting by the surface modification of NPs with specific ligands that can be recognized by transporters (glucose, glutathione, and amino acids transporters) or receptors (transferrin, lactoferrin, LDL, nAChR, and αvβ3 integrin receptors) overexpressed in the brain has been widely used to direct NPs to the desired site of action in the brain. Unfortunately, although there are some undergoing clinical trials, so far, most nanomedicines have not exhibited a significant improvement in therapy efficacy for brain tumors, and thus no nanoformulations have received approval for clinical use. Therefore, there is an urgent need for strategies to develop more advanced multifunctional nanomedicines, which can increase the survival rate of brain tumor patients [44].

### 3.3. Stimuli-Responsive Strategies

As one of the latest advances in nanomedicine, stimuli-responsive systems, also known as smart systems, represent a major advance in the design of nanomaterials capable of gradually releasing their payload, keeping low therapeutic levels of the drug during blood circulation but boosting them when NPs reach targeting sites, after which these levels are maintained.

**Figure 6:**
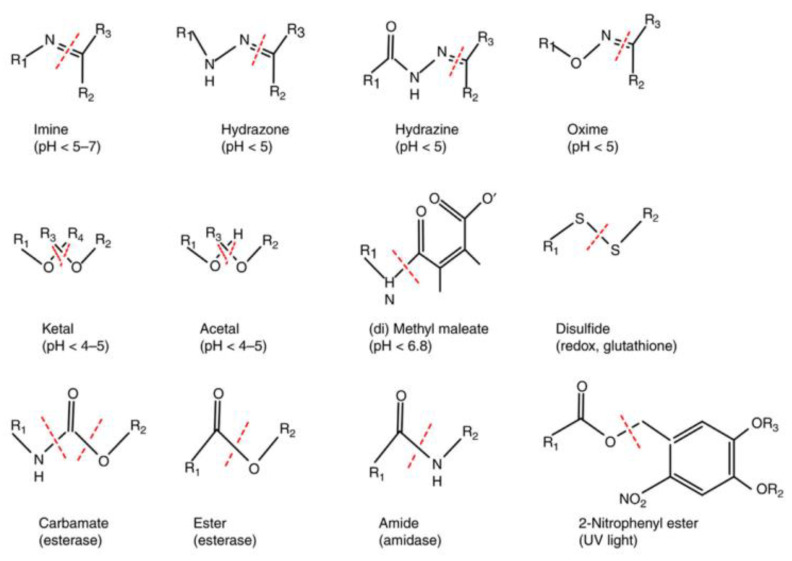
Chemical bonds used to develop stimuli-responsive systems [25]. Reprinted with permission from ref. [25]. Copyright 2017 Elsevier.

Controlled release strategies lead to the development of smart drug delivery systems. This kind of smart system can not only change their structure, morphology, size, and stability, as well as surface properties in response to internal/external stimuli, but can also overcome biological barriers by the reemergence of active targeting ligands via shielding/deshielding transition. As a result, these responsive nanocarriers can activate the drug release at the site of targeted tumors, leading to lower drug toxicity and thus better therapeutic efficiency [5,45,46]. As shown in Figure 6, the main structure in smart nanoplatforms is usually stimuli-responsive polymers that are found as linkers forming part of the skeleton, as polymer conjugates on the outer surface of the nanocarrier, or as polymer–drug conjugates binding bioactive molecules to the nanocarrier [25].

Some different stimuli have been used for controlling drug delivery on the basis of internal and external stimuli. The internal stimuli-responsive nanocarriers can undergo transitions in response to endogenous characteristics or biomarkers such as changes in pH, redox potential, and enzymes, as well as temperature. On the contrary, the external stimuli-sensitive nanocarriers can take advantage of exogenous variables such as light and ultrasound [47,48,49,50]. The internal stimulus-sensitive nanocarriers can provide a relatively universal approach not only for controlled drug release but also for prodrug activation and endosome/lysosome escape [51,52]. The cancer microenvironment is characterized by acidosis and hypoxia due to the presence of lactate; thus, there is a difference between intracellular (6.5–6.8) and extracellular (7.4) pH in cancer cells [47]. The design of the NPs targeted at this mildly acidic TME with the ability to differentiate small pH variations for subsequent delivery of weak basic agents or anticancer drugs, e.g., doxorubicin, improves their accumulation in acidic fluids, and increases the intercellular uptake of drugs and adjusts the drug release within tumor cells from the nanocarriers [14,26,49,50]. In addition to pH-sensitive nanocarriers, hypoxia-sensitive nanocarriers could also be employed, as solid tumors show decreased oxygen levels around them and decrease the effectiveness of a wide range of cancer therapies [53,54]. These nanocarriers are generally made of hypoxia-sensitive materials or their derivatives and are designed to increase the oxygen level of TME through the release of hypoxia-activated prodrugs [55,56].

Abnormal redox balance is one of the characteristics of TME. Glutathione (GSH) is a natural antioxidant whose levels within cancer cells (2–10 mM) are significantly higher than those in healthy cells (2–10 μM) [57], so it can be used as an endogenous trigger for cancer treatments by developing redox-responsive nanomaterials for drug delivery. The latter approach could be applied mainly in combination therapies, as GSH helps modulate and prevent the damage caused by toxic reactive oxygen species (ROS), so the development of GSH-responsive nanoplatforms is necessary to trigger both the generation of ROS and the release of ROS-responsive drugs, thus enabling the disruption of redox self-regulation in cancer cells and an improved therapeutic effect. Disulfide- and diselenide-containing materials are more widely used because of their lower bounding energies and high sensitivity to redox potential [58]. Temperature, light, ultrasound, or magnetic field as typical external stimuli have been widely used to design smart NPs for improved delivery efficacy, which makes it possible to control the location, intensity, and duration of exogenous stimuli so that triggering a controlled release of the payload [59,60,61].

Due to the complicated physiological fluids, complex physiopathological mechanisms, and the high heterogeneity of tumor tissues as well as the abnormal TME, the single stimulus-responsive drug-delivery system often cannot overcome the sequential biological barriers and cannot meet the requirements of complex clinical applications. In the last several decades, there has been a trend to incorporate multiple stimuli to trigger drug release for efficient drug delivery. The multi-modal nanocarriers can take advantage of a mixture of endogenous/exogenous stimuli or can combine diagnostic and therapeutic functionalities into a single nanoplatform. This strategy can not only facilitate NPs to overcome a series of continuous physiopathological barriers in the body, but can also achieve cancer therapies and cancer imaging simultaneously [6,15,48,62,63].

Although many achievements have been made in the development of NP-based chemotherapies, the performance of various NPs is still far from perfect. There are still many challenges that must be addressed to overcome the complex biological barriers and enhance the delivery efficiency of nanomedicine. For example, most traditional nanocarriers, especially liposomes, polymer micelles, and polymer nanogels, always suffer from their low drug loading capacity. The low drug loading content often leads to poor treatment efficacy. In this case, an increase in dosage and/or frequency of administration is required to deliver enough drugs at the specific sites. In addition, these nanocarriers often suffer from an uncontrollable release profile, especially premature drug leakage. Apparently, the premature drug leakage outside the tumor tissue not only causes local or systemic toxicity, but also decreases the bioavailability of nanomedicines and thus restricts the therapeutic efficacy [64,65]. In addition to the undesirable initial burst release, the delayed or inferior drug release within the tumor cells would inevitably cause a very low therapeutic efficacy of nanomedicines, as tumor cells possess an active efflux mechanism and intrinsic drug resistance mechanism to remove intracellular drugs. In addition, the inorganic or metal NPs have exhibited great potential for pharmaceutical applications. However, their applications often suffer from relatively low biocompatibility and relatively high toxicity as well as undesired biodegradation behaviors, limiting clinical translation. Apparently, it is imperative to develop novel and more sophisticated nanocarriers for effective cancer treatment.

## 4. Covalent Organic Frameworks in Antitumor Drug Delivery

In 2005, the group of Yaghi reported the first covalent organic frameworks (COFs) which are produced by use of dynamic covalent boroxine bonds between organic building blocks and linker units [66]. As a kind of new crystalline porous materials, COFs with great potential for various applications have promptly obtained widespread interest, because of their desirable and unique features, such as high porosity, low density, highly tunable pore geometry, intrinsic adaptability, very large surface area, outstanding crystallinity, and excellent flexibility in molecular architecture and functional design [67]. During the last two decades, many COFs with different compositions and structures were made by several techniques using a variety of linkages, including boroxine (-B-O-B-), boronate ester, (-B-O-C-), spiroborate (-B-O-), and borosilicate (-B-O-Si-), and the imide (-C=N-) and hydrazone bond (-C=N-NH-CO-) based on Schiff base reactions. In addition, the β-ketoenamine, phenazine, and triazine linkages were also used to prepare various COFs [68,69]. Due to the availability of organic units and the diversity of topologies and linkages, COFs have emerged as a new field of organic materials. They can not only offer a powerful molecular platform for complex structural design and tailor-made functional development, but also provide a predesignable platform for molecular architecture and materials construction. Apparently, their importance and multifunctionality have endowed COFs with tremendous potential for various applications. As a result, COFs have been widely exploited as promising materials for electrochemistry, electronic devices, gas sorption, storage and separation, organic molecules adsorption, optoelectronics, sensing, pollutant removal, heterogeneous catalysis, environmental remediation, and water treatment. More importantly, COFs have exhibited great potential and opened new revolutionary and intriguing scenarios in biomedical and pharmaceutical fields [70]. Recently, some important reviews have well summarized the structure design and various applications of COFs [71,72,73,74,75]. In this review, the latest applications of functional COFs in anticancer drug delivery to enhance chemotherapy were illuminated.

### 4.1. Unique Properties of COFs as Nanocarriers

As a class of newly emerged crystalline porous polymers, COFs were mainly made from light-weight elements (H, B, C, N, and O) and linked by dynamic covalent bonds such as boronate ester, hydrazone, imine, imide, and azine [67,68,76]. Recently, these porous COFs were reduced to nanoscale particles via mechanical, ultrasonic, or chemical exfoliation. The nanoscale COFs, generally in the form of two-dimensional nanosheets, have opened unprecedented opportunities in cancer therapy, especially for anticancer drug delivery, because a variety of COFs have demonstrated some unique characteristics and desirable benefits when compared with the other porous crystalline materials [77].

These characteristics and advantages mainly include: 1. The high porosity of COFs. COFs, as a class of porous material, not only have a long-range regular pore structure with high tunability, but also possess ordered, continuous, and open channels at the nanoscale. These nanoscale pores and channels of COFs can not only allow for sufficiently loading various guest molecules under non-covalent interactions, but also allow for rapid transport and controlled release without affecting the bioactivity and pharmaceutical effect of such molecules [78,79,80]. Moreover, the very large surface area of COFs can also allow for achieving a high drug-loading content, leading to high therapeutic efficacy and few side effects. 2. The modular nature of COFs structure. The structures of COFs consisted of linkers and spacers with adjustable symmetry and different lengths, and thus the morphology and architecture of the COFs can be readily adjusted and controlled by only the adequate choice of the building blocks at the beginning of the pore design [67,81]. Their customizable organic composition, facile modification of the frameworks’ backbone, and flexible functionalization at the specific binding sites within the structure open a new avenue for the development of environment stimuli-responsive COF-based nanocarriers. 3. The conjugated chains of COFs. Most COFs are crystalline porous materials with π-conjugated structures. On the one hand, this kind of unique chain composition can endow them an improved photocatalytic activity [82], allowing them to react with oxygen to produce reactive oxygen species and participate in the initiation of substrates under photoexcitation [83,84]. On the other hand, the extended π-stacked layers of COFs can offer outstanding conductive channels for charge migration. As a result, some functional COFs derived from organic building blocks with unique optical–electrical properties can be imparted with high electrochemical activity and/or light harvesting capability, allowing for potential applications in the fields of bio-imaging for disease diagnosis or photodynamic photothermal therapy [85,86,87]. More importantly, considering that most chemotherapeutic agents such as doxorubicin, paclitaxel, pirarubicin, and other anthracyclines are aromatic compounds, COFs with π-conjugated structures are desirable for stably loading chemotherapeutic agents by π–π interactions, which has been proven to be a promising and efficient approach to enhance the stability and reduce the burst release. 4. The multifunctionality of COFs. The desired multifunctionalities can be easily incorporated into the skeleton of COFs due to their chemical adjustability. On the one hand, the highly tunable functionalities of COFs can be used to adjust the specific interactions between COFs and guest molecules [88]. On the other hand, the organic backbone of COFs can be functionalized by the introduction of functional groups into specific positions within the framework through both pre- and post-synthetically modification. Generally, COFs used in biomedical and pharmaceutical fields need to be modified by covalently anchoring a series of polymers and biomolecules, such as PEG, lipids, targeting ligands, functional amino acids, peptides, and enzymes. As a result, some new properties, such as stability for long circulation in the blood, environment stimuli responsiveness for controlled drug release, active targeting for site-specific drug delivery [16], chirality [89], and luminescence for biosensor or bio-imaging [90], can be introduced into the walls of the COFs channels. For example, some unique photoelectric performance has been introduced into some COFs by the use of predesigned organic building blocks with photoelectric properties. These functionalized COFs can be used as an ideal theranostic platform due to the combination of the drug therapy and photoelectric diagnostic function in one COF-based system. 5. The high biocompatibility and tunable stability of COFs. Compared with other porous materials, COFs are mainly made from light-weight elements, such as H, B, C, N, and O. The absence of heavy metal ions in their structure is a desirable characteristic for applications such as nanomedicine, as the harmful metals are avoidable and the organic building units could significantly improve biocompatibility and biodegradability [76,91]. In addition, the highly ordered structures were generally formed in COFs due to their π–π stacking interactions, leading to high crystallinity and thus thermal stability [92]. Compared with the liposomes and polymer micelles, COFs are linked by specialized dynamical covalent bonds and their surfaces can be easily modified by the introduction of a hydrophilic polymer chain. As a result, the dispersibility in aqueous solutions and chemical stability during the delivery journey in the body of COFs as a drug delivery system can be well improved [79,93]. Meanwhile, the reversible nature of the crosslinking bonds in COFs endows them with a desirable feature for biodegradability [94]. In sum, compared with conventional materials, the unique structures, desirable properties, and interesting advantages as well as facile functionalization enable COFs to act as a promising nanocarrier for various biomedical applications, especially for anticancer drug delivery [70,95].

### 4.2. COFs for Efficient Drug Loading

As is well known, most chemotherapeutic agents suffer from problems of low solubility and non-specific distribution and poor bioavailability, which limit their applications in conventional cancer treatments. COF-based nanocarriers can serve as a kind of promising and efficient nanoplatform to effectively load drugs, protect drugs from being degraded and prolong the retention time in the body and enhance their delivery and accumulation into tumor sites. The drug-loading strategies of COFs were mainly based on physical adsorption, due to the noncovalent interactions (especially the hydrophobic interaction and π–π stacking interactions) between the functional moieties of hydrophobic therapeutic agents and the pore surface properties of COFs.

Generally, the drug-loading process of COFs is based on incubation within drug solution under vigorous agitation, and the drug-loaded samples were finally obtained after separation by filtration and washing [76]. The drugs are often impregnated in pores and thus the size of drug molecules could fit well with the pore width, which can increase drug loading capacity [96]. In addition, the introduction of nitrogen atoms with the ability to be protonated can exert electrostatic interaction with some drug molecules with electronegative groups, leading to a significant increase in drug loading capacity [76]. Additionally, polyhydroxy drugs can take advantage of hydrogen bonding and van der Waals’ force interaction with oxygen-rich COFs, achieving a high drug-loading capacity [94]. Some studies indicated the amine functional groups of COFs can effectively load various drugs by hydrogen bonding, electrostatic, and van der Waals interactions [97]. In addition, the hydrophobic interactions and π–π stacking interactions are very important for the loading of the aromatic and polycyclic aromatic drugs, such as doxorubicin, paclitaxel, pirarubicin, and anthracyclines [98]. Meanwhile, biomimetic mineralization strategy [99] or core–shell structures have been employed to load bioactive compounds unattainable by traditional means [100,101].

Recently, a novel one-pot synthesis was developed. For example, DOX was successfully encapsulated in situ into COF-based nanocarriers in the preparation process of COFs. This method can not only reduce complicated reaction steps and time-consuming for loading drugs, but also achieve the highest value for existing drug-loaded COFs [102].

### 4.3. COFs for Controlled Drug Release

To achieve high therapeutic efficacy and few side effects, an ideal nanocarrier should be able to show a switchable “off–on” release behavior, that is, tightly retaining the entrapped drug in blood circulation while dumping all the drugs once entering the tumor cells. As mentioned above, COF-based nanocarriers have a very high drug-loading capacity for many chemotherapeutic agents, especially for aromatic and polycyclic aromatic drugs. The premature drug release can be well prevented by appropriate stabilization by some special interactions between drugs and the hydrophobic pores in COFs, such as π–π interactions, hydrogen bonds, and electrostatic complexation. However, the stable COFs–drugs interactions often cause slow drug release at the desired sites, leading to lower efficacy. For example, Fang et al. synthesized two 3D COFs with different pore sizes by choosing tetrahedral building units by use of an imidization reaction between pyromellitic dianhydride (PMDA) and tetrahedral building blocks 1,3,5,7-tetraaminoadamantane (TAA) or tetra (4-aminophenyl) methane (TAPM) at 160 °C, respectively [103]. The obtained COFs were used as drug delivery systems for Ibuprofen, Captopril, and Caffeine. The interpenetrated structure of COFs exhibited a very low release rate in in vitro studies. Bai et al. also reported two different nanoscale porous COFs with good biocompatibility. An anticancer drug, 5-fluorouracil (5-FU), was loaded into the nanoscale COFs. The drug loading capacity, dispersibility in aqueous solution, biocompatibility, and drug release properties were then explored [104]. The 5-FU loading amount was determined by thermogravimetric analysis. The results indicated that both polyimine-based COFs (PI-3-COFs and PI-2-COFs) were proven to be able to achieve a high drug-loading content (up to 30%) due to the drug–COF interactions. The drug-loaded COFs with a size of about 50 nm have very good dispersibility in an aqueous solution. The drug release behavior from PI-3-COFs and PI-2-COFs was real-time monitored by UV-vis spectrophotometry. The drug release rates from PI-3-COFs and PI-2-COFs were found to be almost similar, and 85% of the initially loaded 5-FU were released after 3 days. Although the interactions exert a retardant effect on drug release leading to a slow drug release profile, the porous COFs were demonstrated to be able to serve as smart nanocarriers to efficiently load and slowly release the anticancer drugs [104].

**Figure 7:**
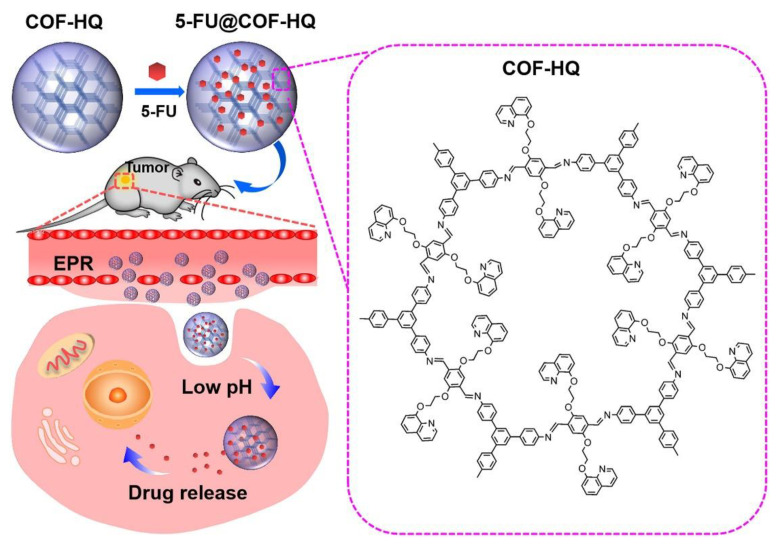
pH-responsive COF-HQ for 5-FU loading and release [76]. Reprinted with permission from ref. [76]. Copyright 2020 Elsevier.

As is well known, the normal human physiological pH is about 7.4, whereas the intratumor microenvironment is acidic because of the accumulation of lactic acid within tumor. The pH of extracellular matrix and intracellular microenvironment of tumor ranges from 4.0 to 6.5. The difference between pH in blood and normal tissues and pH in cancer tissues has been widely used for the specific targeting of tumor and controlled release at the tumor site [49,50,105]. Jia et al. prepared 8-hydroxyquinoline functionalized nanoscale COFs (COF-HQ) by the Schiff base reaction of 1,3,5-tri-(4-aminophenyl)benzene and 2,5-bis (2-(quinolin-8-yloxy)ethoxy)terephthalaldehyde in the presence of acetic acid. The as-prepared COF-HQ showed suitable pore size, stable crystal structure, and pH sensitivity. The anticancer drug 5-FU was loaded into COF-HQ to obtain 5-FU@COF-HQ, as shown in Figure 7. 5-FU@COF-HQ, due to the introduction of the quinoline groups, exhibited excellent dispersibility in the physiological solution. In addition, the presence of numerous conjugated nitrogen atoms derived from the quinoline heterocycle can increase drug loading. 5-FU@COF-HQ was imparted with high drug loading capacity due to electrostatic interactions between the conjugated nitrogen atoms and the strongly electronegative F group in 5-FU. The real-time drug release profiles of 5-FU under different pH conditions were monitored by UV–vis spectrophotometry. A much faster drug release rate at pH 5.0 was observed. This is due to the nitrogen atoms in quinoline groups being fully protonated, and the hydrophobic interaction with 5-FU was destroyed. Considering the acidic TME, 5-FU@COF-HQ with the ability of complete drug release into the tumor can achieve effective suppression of B16 melanoma cells in vitro and in vivo, which can act as a promising platform for 5-FU delivery with high tumor inhibition efficiency [76].

**Figure 8:**
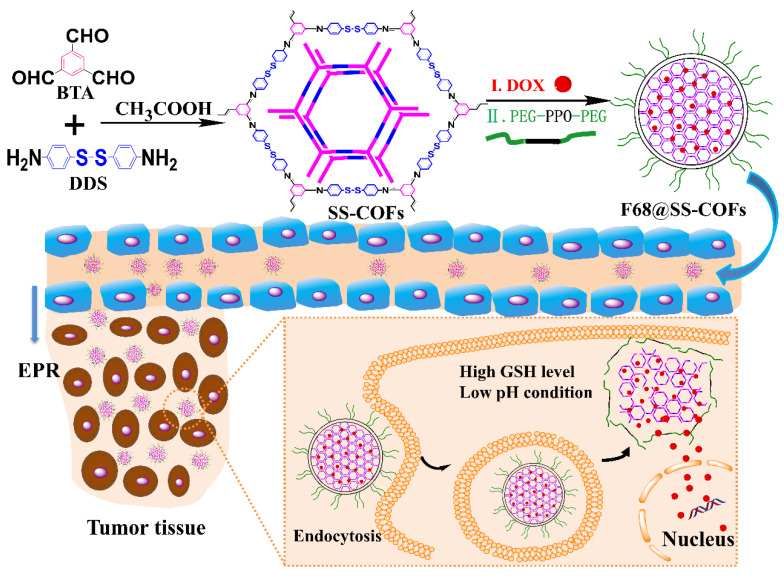
Schematic representation of the preparation and intracellular GSH-responsive drug release of DOX-loaded F68@SS-COFs [106]. Reprinted with permission from ref. [106]. Copyright 2020 John Wiley and Sons.

Compared with the microenvironment of blood circulation and normal tissues, tumors have developed unique TMEs during their evolution. In addition to the acidic TME in tumor tissues and cells, the TME generally exhibits high-level glutathione (GSH), higher hypoxic status, and overexpressed enzymes. Generally, the GSH concentration in tumor cells is reported to be 5–10 mM, which is much higher than that in normal cells or plasma. Therefore, the high level of GSH as specific marker was widely applied for selective drug release in the tumor cytosol by use of redox-responsive disulfide bonds or diselenide bonds in nanomedicine. Recently, Zhang et al. reported a kind of PEGylated redox-responsive COF-based nanocarriers by the self-assembly of Pluronic F68 and disulfide-bearing COFs. The disulfide-bearing COFs were made from commercially available building blocks 4,4′-diaminodiphenyl disulfide and 1,3,5-benzenetricarboxaldehyde (DDS and BTA, respectively) [106]. As shown in Figure 8, the obtained F68@SS-COFs were examined by scanning electron microscope, and the results indicated F68@SS-COFs exhibited spherical morphology with relatively uniform size distribution and particle size in the range of 100–200 nm. The hydrophobic DOX was effectively loaded and achieved a very high drug loading content of about 21% due to the strong hydrophobic and π–π stacking interactions between aromatic rings of nanoscale COFs and DOX. The cytotoxicity and the effect of dilution on the stability of F68@SS-COFs were studied, demonstrating excellent biocompatibility and high long-term extracellular stability. To confirm the GSH responsiveness, the size and morphology change of F68@SS-COFs were investigated and the DOX release profiles were studied in PBS with 10 mm GSH at different times. Due to the disintegration of disulfide moieties, F68@SS-COFs can possess the ability of intracellular controlled drug delivery responding to GSH concentration in tumor cells, resulting in an outstanding growth inhibition [106].

Similarly, they also designed a kind of pH and redox dual-sensitive COFs by the use of a Schiff base reaction between BTA and a new building block (4,4′-Dihydrazide diphenyl disulfide) bearing hydrazide and disulfide bonds. After ultrasound exfoliation and co-assembly with Poloxamer 188, the pH and redox dual-sensitive COF-based drug delivery system (HY/SS-CONs) were prepared and then used to efficiently load DOX. A very high DOX loading content of about 18% was achieved, which was due to the porous structure and high surface area as well as the hydrophobic interactions and π–π stacking interactions between DOX and COFs channels. The dispersion stability during simulative blood fluids of these COF-based nanocarriers with PEGylated surface was investigated. The results demonstrated their excellent stability due to the combined effect of the steady core ensuring integrity of nanostructure and the steric stabilization of PEGylated shell preventing flocculation. Moreover, to confirm the acid and GSH-triggered drug delivery, the size change of HY/SS-CONs and the DOX release profiles were detailed, and investigated in PBS pH 5.0 with 10 mM GSH at different times. The results indicated that the HY/SS-CONs can undergo acid and GSH-triggered structural disintegration within the TME of tumor cells and rapidly release DOX, thus exhibiting a very high inhibition effect for tumor cells [107].

### 4.4. COFs for Targeted Drug Delivery

**Figure 9:**
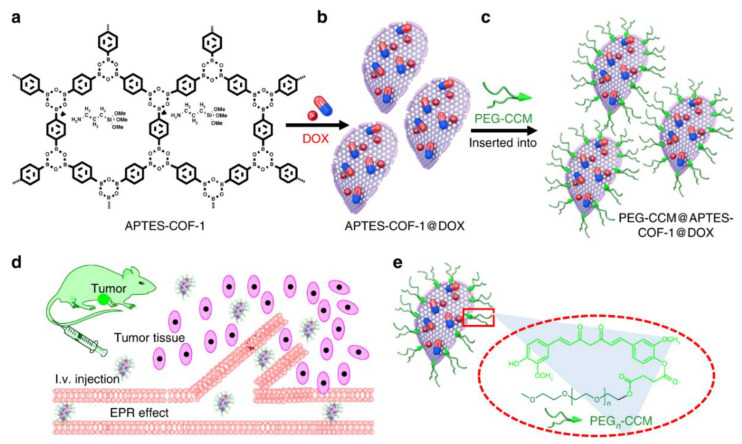
Synthesis of DOX-loaded PEG-CCM@APTES-COF-1 with cellular uptake at weak acidic tumor tissue/cells (**a**–**d**). (**e**) Synthesis of PEG-CCM amphiphilic block copolymers containing hydrophobic blocks based on the unplugging of the PEG-CCM polymer from the APTES-COF-1 pores [108]. Reprinted with permission from ref. [108]. Copyright 2018 Springer Nature.

To achieve high therapeutic efficacy and low side effects, an ideal nanocarrier can not only tightly retain the drug in the system circulation and overcome all biological barriers during the drug delivery to the tumor, but also efficiently and rapidly dump the drug once entering the tumor cells. The improvement of the poor dispersibility of COFs in water and the enhancement of circulation stability of COF-based nanocarriers during the blood circulation system are the first steps toward that goal. The introduction of the PEG layer onto the outer surface COFs can improve the stability of NPs and prolong the blood circulation time. Moreover, PEGylated shells as one of the most effective approaches have been widely used to prevent the occurrence of flocculation and aggregation of NPs. More importantly, PEGylated shells are very important for COF-based nanocarriers to prolong their circulation time and improve drug accumulation in tumors via the EPR effect by enhancing the evasion of recognition by the RES [108]. For example, a series of water-dispersible PEG-COFs nanocomposites (PEG-CCM@APTES-COF-1) as smart nanocarriers for DOX delivery were developed. Firstly, amine-functionalized COFs APTES-COF-1 was synthesized by use of 1,4-benzenediboronic acid in the presence of APTES, as shown in Figure 9a. After DOX was loaded into APTES-COF-1 (Figure 9b,c), the assembly of PEG-modified monofunctional curcumin derivatives (PEG-CCM) and APTES-COF-1 was carried out. The bare APTES-COF-1 was unstable under aqueous physiological conditions, suffering from the formation of large aggregates or a premature release of drugs. However, after modification by PEG-CCM, G-CCM@APTES-COF-1 not only exhibited good and stable dispersion in water at room temperature, but also displayed a very long circulation stability and improved biocompatibility, and biological antioxidant activity. Moreover, in vitro and in vivo studies demonstrate that PEG-CCM@APTES-COF-1 has great potential to be used as a smart carrier for drug delivery, as the results indicated this novel PEGylated COF-based nanocarriers possess superior stability, intrinsic biodegradability, and high DOX loading capacity. More importantly, PEG-CCM@APTES-COF-1 can display a very high fluorescence intensity due to the CCM, allowing for the investigation of the bio-distribution by tracking the intrinsic fluorescence of CCM in organs. Most of the DOX-loaded PEG-CCM@APTES-COF-1 was found to accumulate in tumor tissue via an EPR effect, as the PEG-CCM@APTES-COF-1 exhibited a prolonged circulation in the bloodstream. As a result, DOX-loaded APTES-COF-1 can achieve remarkable anticancer therapeutic efficiency [108].

**Figure 10:**
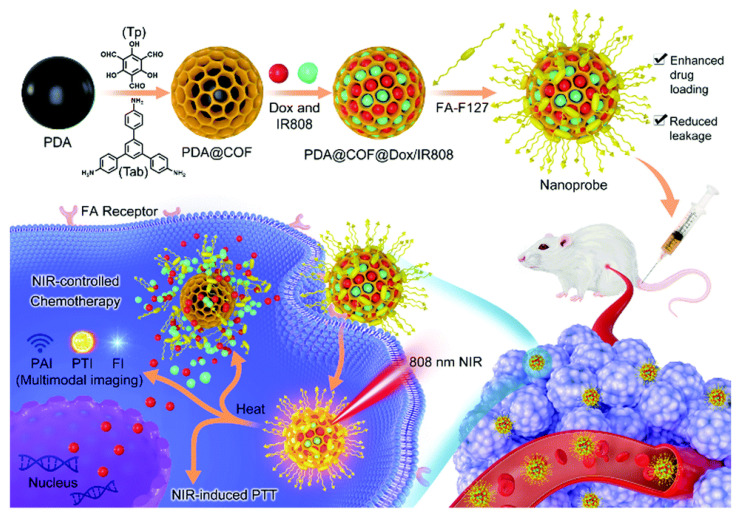
Schematic illustration of the preparation and in vivo multimodal imaging guided photothermal chemotherapy application of the nanoprobe [109]. Reprinted with permission from ref. [109]. Copyright 2021 Royal Society of Chemistry.

Mussel-inspired polydopamine (PDA) was widely applied as biomedical materials and photothermal agents for tumor theranostics owing to its easy preparation, good biocompatibility, and facile modification. Gao et al. reported a COF-coated PDA nanoplatform (PDA@COF) with narrow size distribution for improved drug delivery, as shown in Figure 10. The obtained PDA@COF core–shell nanostructure, as a promising nanocarrier, and the multifunctional tumor theranostic probe can not only achieve a very high DOX loading performance, but can also minimize premature drug release during blood circulation, due to the unique characteristics of the COF shell. After further loading of NIR dye IR808 and coated by folic acid (FA) modified F127 (F127-FA), the final obtained nanosystem (PDA@COF@Dox/IR808@F127-FA, termed as PCDIF) was used as a theranostic probe in NIR fluorescence, photoacoustic, and photothermal imaging, as well as tumor chemotherapy. Due to the FA receptor-mediated uptake, PCDIF could effectively accumulate in the tumor tissue and then be internalized by cancer cells. More importantly, the acidic TME can accelerate the release of DOX. At pH 6.6, 44% DOX was released from PDA@COF@Dox in 8 h, which was much higher than that at pH 7.4. In 8 h, 74% DOX was found released under 808 nm laser irradiation. These results indicated that the PDA cores with good photothermal conversion effect under 808 nm laser irradiation could generate localized hyperthermia, which can be used to further accelerate DOX release and enhance intranuclear DOX accumulation and distribution. In sum, PCDIF can not only achieve an effective DOX loading and delivery, but can also realize a laser-mediated thermal-chemo synergistic therapy under multimodal imaging guidance [109].

The group of Liu designed a new kind of PEG-modified pH-responsive COF-based nanocarriers for combined cancer treatment. Firstly, they used acryloyl chloride to obtain acrylate-conjugated hydrophobic photosensitizer meso-tetra(p-hydroxyphenyl) porphine (acryloyl-THPP). Subsequently, the acryloyl-THPP was used to react with secondary amine 4,4′-trimethylene dipiperidine (TMPD) and mono-amino-terminated PEG (mPEG5k-NH2) via a Michael-addition reaction to form the pH-responsive biodegradable β-amino esters as cross-linkers. DOX was loaded into the THPP–BAE–PEG COFs and then the drug loading and pH-triggered drug release were investigated in the PBS solutions at pH 6.0 and 7.4. The pH-responsive DOX release behavior was demonstrated, and a fast DOX release under pH 6.0 was observed, which can be due to the degradation of β-amino esters under acidic pH. In addition, the obtained DOX-loaded COFs show prolonged blood circulation and efficient tumor-homing ability after intravenous injection. Moreover, the photosensitizer THPP as an organic building block was introduced into the structural framework of COFs. As a result, the THPP-functionalized COFs can not only act as powerful and pH-responsive multifunctional nanocarriers for DOX delivery, but can also induce the death of cancer cells by photodynamic cytotoxicity. That is to say, the designed COFs in this work can readily realize the combined cancer treatment [110].

To improve the transportation ability and bioavailability of COFs, as well as to investigate the fundamental interactions of COFs with living systems, a kind of pristine keto-enamine-linked COFs (TpBD-COFs) was designed and synthesized. The COFs were prepared by the reaction between 1,3,5-triformylphloroglucinol (Tp) and benzidine (BD) under solvothermal conditions. Then the COFs were exfoliated by a metal-assisted aqueous-phase route to produce a kind of ionized COF-based nanosheets with few-layer molecular thickness, uniform belt shape, water solubility, and metal coordination. Moreover, the nanosheets of TpBD-COFs with greatly improved hydrophilicity and bioavailability were demonstrated to achieve an enhanced loading capacity of proteins [93]. The cellular internalization mechanism of protein-loaded TpBD-COFs was investigated, revealing cellular internalization through the clathrin-dependent endocytosis pathway and demonstrating the superiority of low-dimensional nanoscale TpBD-COFs as a protein delivery system into cancer cells.

Currently, there are limitations to the use COF-based nanocarriers for target-specific drug delivery. Mitra et al. developed a facile, salt-mediated synthesis of COFs in the presence of p-toluenesulfonic acid (PTSA) and then they used three sequential post-synthetic modifications to produce target-functionalized COF-based nanosheets [16]. The conjugation of folic acid (FA) in the nanosheet structure was investigated for targeted delivery of 5-FU to breast cancer cells with overexpressed folate receptors. The FA-targeted nanoscale COFs can preferentially deliver the 5-FU to the breast cancer cells through receptor-mediated endocytosis, which can effectively cause the death of cancer cells by the sustained release of the drug. Despite the relatively low drug capacity, the FA-targeted nanosheets with the easy and scalable preparation process, tunable porosity, predesignable functionality, and tunable porosity have demonstrated great potential for the development of a COF-based targeted drug delivery system [16].

Triazine-linked COFs consisting of alternating triazine and phenyl units possessed exceptional porosities, highly chemical stabilities, and rich nitrogen in the triazine rings, which can be used as platforms for effective energy gas storage, drug delivery, and cancer imaging [111,112]. Zhao et al. [113] and Luo et al. [96] studied the feasibility of triazine-linked COFs as drug delivery systems to control the release of IBU in vitro, as the IBU can fit well with the pore width of two networks. In addition, the acid group in IBU can interact with the triazine rings embedded on the pore wall, leading to an improved loading capacity and controlled release of IBU. DOX is highly efficient at inducing apoptosis of tumor cells and has been widely used in tumor chemotherapy. Nevertheless, the DOX often causes severe side effects [114]. In order to avoid the burst release of DOX into the circulatory system and minimize the damage to normal cells, a microporous covalent triazine polymer (CTP) network was synthesized via the Friedel–Crafts reaction between cyanuric chloride and biphenyl in dichloromethane in the presence of anhydrous aluminum chloride as a catalyst. Then the CTP was transformed into nanoscale CTP particles (NCTP) by intense ultrasonication followed by filtration. NCTP with a high surface area was employed as a potential transport system for DOX delivery and controlled release [98]. Due to hydrophobic and π–π interactions, DOX can be effectively encapsulated into the NCTP. The DOX release rate from NCTP was evaluated at pH 4.8 and 7.4 at 37 °C. The DOX release profile showed a rapid release from NCTP at pH 4.8. The dox-loaded NCTP can be effectively uptaken by Hela cells and the fast-release of the DOX in the intracellular space of HeLa cells, leading to the effective killing of cells [98]. Bhaumik’s group used a Schiff base condensation reaction between 2,4,6-triformylphloroglucinol and 4,4′-ethylenedianiline to develop a novel biodegradable nitrogen-containing nanoscale COFs material (EDTFP-1 COFs), which can induce the death of tumor cells, including HCT 116, HepG2, A549, and MIA-Paca2 cells. They also invested the in vitro drug release profiles of EDTFP-1 COFs at different pH levels, 5.5 and 7.4, in a dialysis bag. The pharmacokinetics plot suggested almost no effect of pH in in vitro drug delivery of EDTFP-1 COFs. Their studies indicated that EDTFP-1 COFs with a size of 20–30 nm are suitable for intercellular delivery, which can induce tumor cell apoptosis through the modulation of pro- and antiapoptotic proteins. The authors developed a molecular mechanism. They suggest that the apoptosis mechanism was due to the EDTFP-1 COFs, which can enhance DNA fragmentation and alter the mitochondrial membrane potential. In sum, the EDTFP-1 exhibited great potential to act as an anticancer agent in cancer therapy [91].

The group of Jiang recently reported the first example of hypoxia stimuli-responsive COFs (TA-COFs) by the reaction of 1,3,5-triformyl-2,4,6-trihydroxybenzene and 4,4-azodiaminobenzene. Due to the presence of the azobenzene group (AZO) in the structural framework of TA-COFs, the prepared COFs were imparted with hypoxia-responsiveness. Subsequently, tirapazamine (TPZ) as a hypoxia-activated anticancer prodrug and Chlorin e6 (Ce6) as a photodynamic therapeutic agent were simultaneously coloaded into the above COFs. Then the surface of TA-COFs was decorated by mPEG5k-NH2 by reaction with aldehyde groups at the COF surface to prolong their circulation time in the blood. The TA-COFs were selectively deintegrated in tumor sites due to the high expression of azo reductase within cancer cells and the agents loaded were selectively released to tumor tissue. At the same time, Ce6 loaded in the COFs can generate ROS under light exposure to specifically kill cancer cells and at the same time cause tumor hypoxia. Moreover, the hypoxic microenvironment induced by Ce6 under light exposure can accelerate the deintegration of TA-COFs, leading to an enhanced TPZ release and thus an increased toxicity of TPZ. Consequently, Ce6 and TPZ coloaded COFs can act as promising light-activated hypoxia-sensitive drug delivery nanoplatforms with synergistic anticancer efficiency in tumor cells and tissue [115].

## 5. Conclusions

This work systematically examined the recent advances of COF-based nanocarriers for anticancer drug delivery. Despite the encouraging outcomes, the COF-based nanocarriers are still at the initial stages. Their successful clinical translation still remains a formidable challenge. Firstly, COFs are generally crystalline porous solids. They are often insoluble and processable solids. As a result, the controllable fabrication of nanoscale COFs with desirable size and morphology is often very difficult. In addition, the poor dispersibility of COFs materials and related COFs particles in aqueous solution urgently need to be addressed. Although the surface modification with PEG and other similar hydrophilic polymers has demonstrated its success, there are few reports about COF-based nanocarriers with controlled morphologies and advanced functionalities rendering them appropriate for clinical use. Furthermore, for the manufacture of COFs and related nanocarriers, the requirement of tedious synthetic steps and toxic reagents makes it very difficult to achieve a large-scale fabrication of COF-based nanocarriers with high quality. Moreover, the controllable and reproducible synthesis of COFs under ambient conditions still faces a number of issues needed to be addressed. Second, COF-based nanocarriers have a remarkable drug loading capacity, especially for the widely used chemotherapeutic agents such as doxorubicin, paclitaxel, pirarubicin, and other anthracyclines that are aromatic compounds. However, in vivo applications of COF-based nanocarriers still need to address several important challenges for effective drug delivery and controlled drug release within the targeted tumor cells. Third, the biocompatibility and toxicity of COFs are crucial for their pharmaceutical applications. Currently, most work on this topic only focuses on improving delivery efficacy rather than alleviating toxicity of COF-based nanomedicine. In addition, few works were done to resolve the challenges in the biodegradability and biocompatibility of COFs, especially lacking a detailed investigation on the metabolism pathways and biodistribution of COF-based nanocarriers. Finally, although the therapeutic potential of COF-based nanomedicine has been demonstrated, the delivery of sufficient amounts of anticancer drugs in the targeted disease site is still challenging. The unintended accumulation in normal tissues often leads to severe side effects for patients. Therefore, how to endow COF-based nanomedicine with the ability to target delivery is a key issue to be addressed. Although significant efforts have been focused on improving their targeting delivery ability by use of active and passive targeting strategies, no significant improvement has been achieved. Future work should systematically reinvent their physicochemical properties to overcome the multiple biological barriers of complex tumor heterogeneity. Apparently, the multifunctional COF-based nanocarriers have exhibited significant advantages for anticancer drug delivery. However, there is still a long way to go to achieve clinical applications. Thus, collaborative and multidisciplinary research efforts are still needed to achieve more successful applications of COFs in pharmaceutical science.

## Figures and Tables

**Table 1:** Nanomedicines available in the market for cancer treatment.

Nanomedicines	Nanoformulations	Drug	Oncology
Oncaspar^®^ commercialized by Enzon in 1994	PEGylated enzyme	L-asparaginase enzyme	Acute lymphocytic leukemia
Doxil^®^ commercialized by Johnson and Johnson in 1995	Liposome	Doxorubicin	Kaposi’s sarcoma, ovarian cancer, breast cancer, multiple myeloma
DaunoXom^®^ commercialized by Galen in 1996	Liposome	Daunorubicin	Kaposi’s sarcoma
Lipodox^®^ commercialized by Taiwan Liposome in 1998	Liposome	Doxorubicin	Kaposi’s sarcoma, breast cancer, ovarian cancer
Ontak^®^ commercialized by Eisai in 1999	Protein-based formulation	Denileukin diftitox	Cutaneous T-cell lymphoma
DepoCyt^®^ commercialized by Pacira in 1999	Liposome	Cytarabine	Neoplastic meningitis
Myocet^®^ commercialized by Cephalon in 2000	Liposome	Doxorubicin	Breast cancer
Eligard^®^ commercialized by Tolmar in 2002	Polymeric nanosuspension	Leuprolide acetate	Prostate cancer
Abraxane^®^ commercialized by Abraxis in 2005	Albumin based NPs	Paclitaxel	Breast cancer, pancreatic cancer, lung cancer
Oncaspar^®^ commercialized by Enzon-Sigma-Tau in 2006	Polymer protein conjugate	L-asparaginase	Leukemia
Genexol-PM^®^ commercialized by Samyang in 2007	PEG-PLA polymeric micelle	Paclitaxel	Breast cancer, lung cancer, ovarian cancer
Mepact^®^ commercialized by Takeda in 2009	Liposome	Mifamurtide	Osteosarcoma
NanoTherm^®^ commercialized by Magforce in 2010	Metallic NPs	Iron oxide	Glioblastoma
Marqibo^®^ commercialized by Talon in 2012	Liposome	Vincristine	Acute lymphoid leukemia
Onivyde^®^ commercialized by Merrimack Pharma in 2015	Liposome	Irinotecan	Pancreatic cancer
Vyxeos^®^ commercialized by Celator in 2017	Liposome	Cytarabine	Acute myeloid leukemia
Hensify^®^ commercialized by Nanobiotix in 2019	Metallic NPs	Hafnium oxide	Locally-advanced soft tissue sarcoma

## Data Availability

Not applicable.
